# Human papillomavirus genotype profiles and cytological grades interlinkages in coinfection with HIV

**DOI:** 10.11604/pamj.2020.35.67.21539

**Published:** 2020-03-10

**Authors:** Lucy Wanja Karani, Stanslaus Musyoki, Robert Orina, Anthony Kebira Nyamache, Christopher Khayeka-Wandabwa, Benuel Nyagaka

**Affiliations:** 1 School of Health Science, Kisii University, Kisii, Kenya; 2 Department of Microbiology, Kenyatta University, Nairobi, Kenya; 3 School of Pharmaceutical Science and Technology (SPST), Health Science Platform, Tianjin University, Tianjin 300072, China

**Keywords:** Human immunodeficiency virus, human papillomavirus, co-infection, genotype, cervical carcinoma, pathology, vaccine

## Abstract

**Introduction:**

The study aimed to examine and characterize human papilloma virus (HPV) cytological grade trends and genotypes among HPV/HIV co-infected/cases and HPV monoinfected/control women attending Kisii Teaching and Referral Hospital, Kenya.

**Methods:**

HIV positive co-infected with HPV (HPV/HIV) and HIV negative women monoinfected with HPV profiled as co-infected/cases and monoinfected/control arms respectively were enrolled. HPV subtypes were assayed by Xpert^®^ HPV system (GXHPV-CE-10) alongside pathological cytology analysis of cervical tissue samples.

**Results:**

Low grade intraepithelial lesion (LSIL) was the most predominant cytological grade across cases and controls with a prevalence of 32 (38.1%) while high grade squamous intraepithelial lesion (HSIL) was highest among HPV/HIV co-infected with a prevalence of 23 (27.38%). Among the monoinfected (controls) the predominant lesion was low grade intraepithelial lesions (LSIL) with a prevalence of 23 (27.38%). HPV type 16 had the highest prevalence 26 (30.8%) among the VIA positive women in the overall study participants followed by combinations of HPV types (16, 18/45) at 19 (22.6%).

**Conclusion:**

High risk HPV types 16 and 18/45 were the most predominant in the established cytological grades and among the co-infected women. Routine screening using both cytological and HPV testing should be embraced and/or reinforced as early screening and preventive strategies in the covered geographical region population. Provision of the currently available vaccines to these women at an early age would provide effective protection since the HPV type profiles in this population are covered by such vaccines.

## Introduction

Human Papillomavirus (HPV) is classified as high risk or low risk depending on its association with infection progression to cancer [1]. Human Immunodeficiency Virus (HIV) infection among women already infected with HPV infection has been correlated to having an association with a greater prevalence with broader ranges of the high risk human papilloma virus (HR-HPV) genotypes [1-3]. The presence of HIV is thought to increase the risk and susceptibility to infections with oncogenic human papilloma virus (HPV) which subsequently accelerates the natural history of Invasive Cervical Carcinoma (ICC) [2, 3]. Cytological findings point to the fact that there is greater rate of persistence in high risk HPV type infections in HIV infected women leading to high-grade squamous intraepithelial lesions (HSIL) which culminate to invasive cervical carcinoma [4]. Infections with HIV is thought to be responsible for alteration of the spectrum of HPV types in normal cervical epithelium as well as in invasive cervical carcinomas resulting to a complicated morbidity in the interplay between the two viruses [5].

According to data at Kenya Medical Research Institute (KEMRI) which serves as regional cancer registry, approximately 80% of reported cases of cervical carcinoma are diagnosed at advanced stages, when little can be achieved in terms of curative treatment [6, 7]. Even with HIV infection and cervical cancer co-infections being a major public health concern, molecular characterization of HPV types and trends specific data in HIV/HPV co-infections are scarce. In Kenya, to the best of our knowledge there is no such data in Kisii region as part of the broader Nyanza geographical terrain. Such gaps in the co-infections and genotype dynamism, translates to many setbacks in screening and prophylactic vaccination strategies among the vulnerable groups [4, 8, 9]. Studies have recommended further research in deciding the most appropriate screening approaches for different populations [2, 8, 9]. In order to better predict the impact of current vaccines on cervical cancer and for the improvement of screening programs, regional data on distribution of human papillomavirus (HPV) types in women with or without cervical cancer is crucial [10, 11]. Thus, in view of the evidence gap, the study aimed to examine and characterize HPV cytological grade trends and genotypes among VIA positive HPV/HIV co-infected and HPV monoinfected women attending Kisii Teaching and Referral Hospital, Kenya in a quest to generate and enhance regionally adapted insights for better public health cues and planning information.

## Methods

**Study design and setting**: the case-control study was conducted at Kisii Teaching and Referral Hospital (KTRH) located in Kisii County, Kenya. The hospital being a referral hospital receives between 1400-2000 HIV patients per month with a population of over 900 HIV positive women of reproductive age (Hospital records, 2017). The reproductive Health clinic attends to an average of 300 women of reproductive age per month with an average of 80 women of reproductive age being HIV positive (Hospital records, 2017).

**Sample size and participants:** the study targeted women within reproductive age, between 18-59 years who attended the reproductive health care clinic at KTRH between July 2018 and July 2019 who met the inclusion criteria. The age bracket was also within the prevalence peak period when most reproductive age women are sexually active [9, 12, 13].

**Inclusion criteria**: all consented sexually active women between 18-59 years of age; non pregnant women; no previous diagnosis of cervical cancer; had not used vaginal medication for the previous two days; HIV positive women with HPV infections for cases and HIV negative women with HPV infections for controls.

**Exclusion:** those who failed to give written consent; pregnant women; women who are < 18 and > 59 years; women with other medical conditions such as mental incompetence, had hysterectomy, or had treatment on the cervix less than 6 months prior to visit or on menstruation; HIV positive women without HPV infections and HIV negative women without HPV infections. According to needs assessment data obtained from Kisii Teaching and Referral Hospital records in the year 2017, a total of 4676 women were screened for cervical cancer using Visual inspection with Acetic Acid and Visual Inspection with Lugol's Iodine (VIA/VILI). Out of those screened, 846 (18%) were VIA/VILI positive but HIV negative and 2151 (46%) were HIV and VIA/VILI positive (Hospital records, 2017). In reference to a clinical study in Kenya, the prevalence of HPV amongst HIV infected women was 49% while HPV prevalence in HIV negative women was 17% [14]. Going by such prevalence and underpinnings, we arrived at an independent case control study with HIV positive women co-infections with HPV as cases and HIV negative women monoinfected with HPV as controls [15, 16] while adopting the double proportion formula in arriving at adequate sample size [15]. The minimum required sample size was therefore 32.675; 33 study participants for each group/arm making a total minimum of 66 participants for the two groups/arms.

The study participants were recruited by invitation. Three female nurses and one female clinical officer with prior knowledge and experience on visual inspection of the cervix with acetic acid (VIA) were trained on recruitment process of the participants and how to obtain satisfactory cervical smears. They also worked in conjunction with a residence gynecologist who oversaw routinely the process of sample collection as is recommended [17]. A research assistant was also trained to consent the participants per the bioethically approved study protocol. After consenting, the women underwent visual inspection of the cervix using 5% acetic acid so as to pick the VIA positive clients[18, 19]. The VIA positive women had their cervical smear samples collected for both cytology and HPV genotyping. Additional laboratory and clinical data such as other infections and pregnancy test results was obtained from enrolled participants medical records at the facility. All qualified participants’ data were coded and anonymized.

**Cervical specimens for cytology and High-Risk (HR) HPV genotyping:** all VIA positive women had cervical cell samples exfoliated and preserved for confirmatory testing with Pap and HPV genotyping [20-24]. Briefly samples of cervical cells (cervical swabs) were exfoliated from the zone of transformation on the cervix were carefully exfoliated [22, 23] To guarantee sampling of adequate cells for both cytology and genotyping a cervical broom-like device (Rovers Medical Devices, Netherlands) designed to make contact with ectocervix and endocervix simultaneously was carefully used [20, 21]. Immediately after exfoliation of the cervical cells, a thin smear was made on the microscope slide using the broom and fixed for cytology [18, 19, 25]. The cervical cells left on the broom after the smear preparation on slide were immediately collected in PreservCyt^®^ Solution (Hologic Corp.) per manufacturer protocols [26-29]. There was no need of multiple sample collection or multiple visits since Food and Drug Administration (FDA) approves one Thin Prep Pap Test as the only liquid based technique for both Pap and HPV testing directly [24]. The Pap samples were prepared in duplicate to allow two pathologists to read them independently via microscopy as earlier detailed [22, 23, 30, 31]. The cytology cells slides were classified, categorized and reported according to the revised standardized 2001 Bethesda classification [31, 32]. The women with VIA negative screening results were advised to repeat VIA screening once in every three years as is recommended [18, 19].

For HR-HPV genotyping, all samples were analyzed by Xpert^®^ HPV assay system (GXHPV-CE-10). Based on the manufacturer's instructions, samples were shaken for several seconds, and 1 ml total volume of PreservCyt was poured into an Xpert^®^ HPV cartridge and subsequently loaded into Cepheid Xpert Diagnosis instrument that uses a second generation real-time PCR [27, 28]. Interpretation of assay results followed the manufacturer's guidelines as previously described and per the recommended WHO standards [26-29]. The applied Xpert^®^ HPV assay specifically identifies types HPV 16 and HPV 18/45 in two distinct detection channels, and reports 11 other high risk types (31, 33, 35, 39, 51, 52, 56, 58, 59, 66 and 68) in a pooled result [26-29]. The 14 specific high risk HPV genotypes were characterized: 16, 18, 31, 33, 35, 39, 45, 51, 52, 56, 58, 59, 66 and 68 with some expressed as pooled results in this diagnostic kit as follows; P1 color channel HPV 16 was detected, P2 color channel HPV 18/45 pooled result was detected, P3 color channel pooled result of any of HPV types 31, 33, 35 52, or 58 was detected, P4 color channel pooled result of either of HPV types 51 or 59 was detected and P5 color channel pooled result of any of the HPV types 39, 56, 66 or 68 was detected. Assay results were reported as negative or positive for HPV16, HPV 18/45, or other HPV types. A Probe Check Control and a Sample Adequacy Control were included in each cartridge [26-29].

HIV testing was done at the reproductive health clinic at KTRH according Ministry of Health, National AIDS & STI Control Program (NASCOP) 2018 national testing guidelines using the Determine^®^ rapid test kit (Abbot Pharmaceuticals, Chicago, USA), and the positive results were confirmed by Uni-Gold^®^ (Trinity Biotech Plc, Ireland). Those found to be HIV positive were counselled and referred to the relevant unit for initiation of treatment.

**Data management and analysis:** the data collected was cleaned, coded, and entered into a Microsoft Excel database developed for this purpose. Statistical analysis was performed using SPSS software, version 20. Cervical lesions and HPV characterization data was analysed using categorical variables which were summarised by descriptive statistics using prevalence and the significance of their difference tested by one sample chi-square statistics. The significance level was set at P < 0.05.

**Ethics approval and consent to participate:** ethical clearance was obtained from the Registrar, Research and Extension Kisii University, Scientific Ethical Committee of University of Eastern Africa Baraton, National Commission for Science, Technology and Innovation (NACOSTI) and Research Authorization given from the office of County Director of Education Kisii County. Written informed consent was given by participants who consented to participate in the study.

## Results

**Figure 1:**
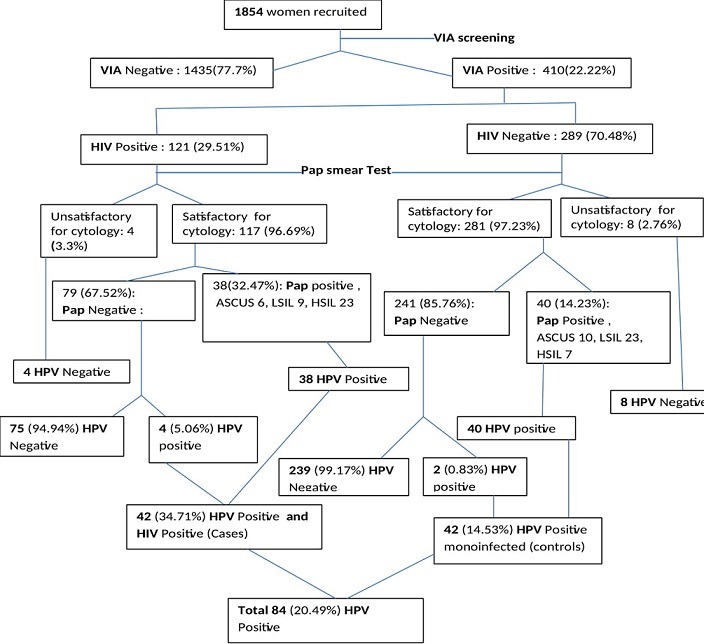
Clinical characteristics and screening of eligible participants in recruitment from preliminary stage to desired sample size

**Clinical characteristics of the participants:** clinical characteristics and screening of eligible participants in recruitment from preliminary stage to desired sample size attainment in the enrollment process are as shown in Figure 1. The enrollment of participants was purposeful and consecutive where 1854 consented participants were recruited before the required sample size could be achieved. The women first underwent Visual Inspection with 5% Acetic Acid (VIA) where 410 (22.22%) women were VIA positive. All the 410 VIA positive samples qualified for cytological analysis and HPV testing. VIA/ HIV positive women were 121 in number and all of them had cervical cell samples exfoliated and smears prepared on slides for cytological evaluation and another sample for HPV characterization. Out of the 121 samples, 117 (96.69%) samples/slides were satisfactory for cytology. The acceptable cytology samples had 79 (67.52%) women with normal cytology and 38 (32.32.47%) had abnormal cytology. There were 289 VIA positive/HIV negative women. All of them had cervical cell samples exfoliated and smears prepared on slides for cytological evaluation of which, 281 (97.23%) were satisfactory for cytology. The suitable samples had 241 (85.76%) women with normal cytology while 40 (14.23%) had abnormal cytology. All the unsatisfactory samples from VIA/HIV positive 4 (3.3%) and those of VIA positive/HIV negative 8 (2.76) were also HPV negative. In the category of women with abnormal cytology, 38 (32.47%) VIA/HIV positive and 40 (14.23%) VIA positive/HIV negative women were HPV positive while only 4 (5.06%) with normal cytology from the VIA/HIV positive and 2 (0.83%) of VIA positive/HIV negative were HPV positive. There was a significance difference (P = 0.029) in the population of women required to recruit the adequate sample size of cases (121) compared to that of controls (289), with controls requiring more than twice the population of cases (Table 1). Thus in the recruitment process one arm had 42 (34.71%) HIV positive women co-infected with HPV/ with a mean age of 40.36±11.318 years while the control arm had 42 (14.53%) HIV negative women who were monoinfected with HPV with a mean age of 35.21±9.495 years.

**Table 1:** Recruitment summary of the cases and controls from 410 VIA positive women

HIV Status	HPV +VE	HPV –VE	Total	P value
HIV +VE	42	79	121	0.029
HIV-VE	42	247	289	
Total women	84	326	410	

**Cytological grades analysis and prevalence:** the 410 samples that were VIA positive were evaluated for cytological grading, 398 (97.07%) had satisfactory smears for cytology which were evaluated for cytological abnormalities. The overall cytological categories in both co-infection (HPV/HIV) and monoinfections (HPV) were analyzed, graded and classified according to the revised standardized Bethesda 2001 [31, 33]. These included a typical squamous cell of undetermined significance (ASCUS), low grade intraepithelial lesions (LSIL) and high grade intraepithelial lesions (HSIL). The cytological grades established ranged from LSIL as the most prevalent followed by HSIL and ASCUS while normal cytology was the least observed cytology across cases and controls (Table 2). There were significant differences (p<0.05) in the frequencies of cytological grades between the HIV positive women (cases) and HIV negative women (controls) (Table 2). The cytological grade with the highest frequency among HIV positive (cases) was HSIL 23 (27.38%) while the least frequency in the same group was normal 4 (4.76%). The cytological grade with the highest and least frequency among HIV negative (controls) was HSIL 23 (27.38%) normal 4 (4.76%) in that order.

**Table 2:** Cervical cytological grades in HPV infected women

Cytological grade	Frequency	HIV +VE	HIV –VE	P- Values
NORMAL	6 (7.10%)	4 (4.76%)	2 (2.38%)	0.025*
ASCUS	16 (19.0%)	6 (7.14%)	10 (11.90%)	0.09
LSIL	32 (38.1%)	9 (10.71%)	23 (27.38%)	0.002*
HSIL	30 (35.7%)	23 (27.38%)	7 (8.33%)	0.006*
Total	84 (100%)	42	42	

**HPV genotypes:** whereas there was no significance difference in the prevalence of HPV type's combinations (16) and (16, 18/45) between HPV/HIV co-infections and HPV monoinfections p = 0.067 and 0.05 respectively, all the other type combinations had significant difference between HPV/HIV co-infections and HPV monoinfections P < 0.05 (Table 3). The HPV type 16 had the highest prevalence 26 (30.8%) in the overall study population while the combination of HPV types (16, 18/45) was second most prevalent with 19 (22.6%) cases. HPV types 18/45 had a prevalence of 11(13.1%). The most prevalent HR-HPV type among both HPV/HIV co-infected and HPV monoinfected women was type 16 at 14 (16.67%) and 12 (14.29%) cases respectively. The most common HR-HPV type combinations in HPV/HIV co-infections were (16), (16,18/45), (16,18/45, P3{31, 33, 35 52, or 58}) and (18/45) with 14 (16.67%), 10 ( 11.90%), 5 (5.95%) and 4 (4.762%) respectively. The most prevalent HR-HPV type combinations in HPV monoinfections were (16), (16, 18/45), (18/45) and (18/45, P4 {51 or 59}) with 12 (14.29%), 9 (10.71%), 7 (8.33%), 7 (8.33 %) respectively.

**Table 3:** HPV genotypes variations among HPV/HIV co-infected cases and HPV mono-infected control women

	HIV status				
HPV types	HIV +ve n=42	HIV –ve n=42	Proportion	P-value
16	14 (16.67)	12 (14.29)	31.0	0.067
16,18/45	10 (11.90)	9 (10.71)	22.6	0.050
16,18/45,P3	2 (2.38)	0 (0.00)	2.4	< 0.001*
16,18/45,P3	5 (5.95)	0 (0.00)	6.0	0.006*
16,18/45,P3,P5	1 (1.19)	0 (0.00)	1.2	0.005*
16,P3,P5	1 (1.19)	0 (0.00)	1.2	0.0002*
16,P5	1 (1.19)	0 (0.00)	1.2	0.0002*
18/45	4 (4.76)	7 (8.33)	13.1	0.0003*
18/45,P3	2 (2.38)	0 (0.00)	2.4	0.0081
18/45,P3,P4	1 (1.19)	0 (0.0)	1.2	<0.001*
P3	1 (1.19)	0 (0.00)	1.2	0.0003*
16,18/45,P4	0 (0.00)	4 (4.76)	4.8	0.0002*
18/45,P4	0 (0.00)	7 (8.33)	8.3	0.0001*
P4	0 (0.00)	2 (2.38)	2.4	<0.00013*
16,P4	0 (0.00)	1 (1.19)	1.2	0.005*
**Total**	**42 (100)**	**42 (100)**	**100.0**	

P denotes Pooled types categories of HR-HPVs; P3 are types (31, 33, 35 52 or 58), P4 (51 or 59) and P5 (39, 56, 66 or 68)

**HPV genotypes characterization across all cytological grades of cases and controls:** the overall HR-HPV prevalence in VIA positive women was 84 (20.49%) with HR-HPV prevalence being high among women with abnormal cytology 34% compared to normal cytology 1.46%. In relation to HIV status, the cytological grade that had the highest HPV prevalence was low grade squamous intraepithelial lesions (LSIL) in controls with type categories (16) and (16, 18/45) each category having a prevalence of 5 (6.0%). In HSIL of controls the HPV type (16) had the highest prevalence 14 (16.67%) followed by HPV type category (16, 18/45) with a prevalence of 6 (7.14%). In cases, HSIL cytological grade had second highest total HPV prevalence of 30 (35.7%) in type categories 16 with a prevalence of 9 (10.7%) followed by type categories (16, 18/45) with a frequency of 6 (7.1%). In these cytological grades the most prevalent HPV types were (16) and (16, 18/45). This study shows an increase in prevalence of HPV type 16 from normal cytology to LSIL and HSIL having 4 (4.8%), 6 (7.2%) and 14 (16.6%) respectively. ASCUS grade in co-infections was having a combination of HPV types (16, 18/45) with a prevalence of 5 (6.0%). With increase in severity of cervical lesions, HPV 16 takes the dominance with a prevalence of 14 (16.6%) followed by (16, 18/45) combinations with a frequency of 6 (7.1%). It was noted that in the cytological grades, type combinations (16, 18/45, P3 {31, 33, 35 52 or 58}, P4 {51 or 59}), (18/45), (16, 18/45, P3 {31, 33, 35 52 or 58}) with a prevalence of 4 (4.8%) and 2 (2.4%) respectively had types 35 and 52 appearing quite often in the early cytological grades. Other types that appeared adversely in the advanced grades include (16, 18/45, P3{31, 33, 35 52 or 58}, P4 {51 or 59}), (18/45), (16,18/45, P3 {31, 33, 35 52 or 58}), (16,P5 {39, 56, 66 or 68} {39, 56, 66 or 68}), (18/45,P3 {31, 33, 35 52 or 58},P4 {51 or 59}), (18/45,P4 {51 or 59}) with prevalence of 4 (4.8%), 2 (2.4%), 1 (1.2%), 1 (1.2%), (1.2%) and (1.2%) respectively. All the other HPV type prevalence in the cytological grades by HIV status together with their p- values indicating whether there is a significance difference between cases and controls are as profiled in Table 4.

**Table 4:** HPV genotypes characterization across all cytological grades of cases and controls

HIV status	HPV types	Cytological grades				Total	P-Value
		NORMAL n (%)	ASCUS n (%)	LSIL n (%)	HSIL n (%)		
**+VE (Cases)**	16	2 (2.4)	2 (2.4)	1 (1.2)	9 (10.7)	14 (16.6)	0.0002
**-VE (Controls)**	16	2 (2.4)	0 (0.0)	5 (6.0)	5 (6.0)	12 (14.2)	
**+VE (Cases)**	16,18/45	0 (0.0)	1 (1.2)	3 (3.6)	6 (7.1)	10 (11.9)	0.001
**-VE (Controls)**	16,18/45	0 (0.0)	4 (4.8)	5 (6.0)	0 (0.0)	9 (10.7)	
**+VE (Cases)**	16,18/45,P3	0 (0.0)	0 (0.0)	1 (1.2)	1 (1.2)	2 (2.4)	0.444
**+VE (Cases)**	16,18/45,P3,P4	0 (0.0)	0 (0.0)	1 (1.2)	4 (4.8)	5(6.0)	0.333
**+VE (Cases)**	16,18/45,P3,P5	0 (0.0)	1 (1.2)	0 (0.0)	0 (0.0)	1 (1.2)	<0.0001
**+VE (Cases)**	16,P3,P5	0 (0.0)	0 (0.0)	1 (1.2)	0 (0.0)	1 (1.2)	<0.0001
**+VE (Cases)**	16,P5	0 (0.0)	0 (0.0)	0 (0.0)	1 (1.2)	1 (1.2)	<0.0001
**+VE (Cases)**	18/45	2 (2.4)	1 (1.2)	0 (0.0)	1 (1.2)	4 (4.8)	0.282
**-VE (Controls)**	18/45	0 (0.0)	2 (2.4)	4 (4.8)	1 (1.2)	7 (8.3)	
**+VE (Cases)**	18/45,P3	0 (0.0)	0 (0.0)	2 (2.4)	0 (0.0)	2 (2.4)	0.444
**+VE (Cases)**	18/45,P3,P4	0 (0.0)	0 (0.0)	0 (0.0)	1 (1.2)	1 (1.2)	<0.0001
**+VE (Cases)**	P3	0 (0.0)	1 (1.2)	0 (0.0)	0 (0.0)	1 (1.2)	<0.0001
**-VE (Controls)**	16,18/45,P4	0 (0.0)	2 (2.4)	2 (2.4)	0 (0.0)	4 (4.8)	0.14
**-VE (Controls)**	18/45,P4	0 (0.0)	2 (2.4)	4 (4.8)	1 (1.2)	7 (8.3)	0.282
**-VE (Controls)**	P4	0 (0.0)	0 (0.0)	2 (2.4)	0 (0.0)	2 (2.4)	0.444
**-VE (Controls)**	16,P4	0 (0.0)	0 (0.0)	1 (1.2)	0 (0.0)	1 (1.2)	<0.0001

P denotes Pooled types categories of HR-HPVs; P3 are types (31, 33, 35 52 or 58), P4 (51 or 59) and P5 (39, 56, 66 or 68)

## Discussion

The study examined and characterize HPV cytological grade trends and genotypes among VIA positive HPV/HIV co-infected and HPV monoinfected women attending Kisii Teaching and Referral Hospital, Kenya in a quest to generate and enhance regionally adapted insights for better public health cues and planning information for instance thus, bringing to the fore understandings that would prove valuable in deciding on the most appropriate screening strategies and HPV vaccination programs approach. Overall, there was 27.38% prevalence of high grade squamous intraepithelial lesions (HSIL) among the HIV positive HPV co-infected women which was the predominant lesion. The overall prevalence of HR-HPV in the VIA positive women in this population was 20.48% while the highest prevalence of high risk HPV genotypes was type 16 while genotypes (16), (18/45) and (16, 18/45) which accounted for 66.67% of the cases. In the cytological grading of participants who were HR-HPV positive regardless of HIV status, the prevalence increased in the order; f normal cytology, ASCUS, HSIL to LSIL albeit, there was marginal difference between LSIL and HSIL cytological grade prevalence. There was diverse variance in prevalence trends of the predominantly prevalent HPV types in the cytological grades ASCUS, LSIL, HSIL across numerous findings reported this far but outstandingly, normal cytology was the least observed cytological grade in regard to HR- HPV infection [34-36]. It has been reported, that the age of the participant, history of other infections and number of pregnancies increases the severity of dysplasia and HPV prevalence [36] thus the most probable factors that cause the difference in dysplasia in these studies.

Related findings have also noted that HPV types did not influence severity of dysplasia rather the duration of HPV infection and immunological responses to infections by the host determine cervical tissue transformation to ICC after HPV infections [36]. There could also be differences in the natural history of the infections in women above reproductive age since the immunological responses to infections, inflammation and anti-cancer responses are reduced with increase in age [37]. The aspect of geographical disparities and ethnicity may also contribute to these discrepancies in cytological grades as shown by differences in genotype specific HPV in some populations from different regions and with differences in ethnicity inclination [38] whereas differences in prevalence of HR-HPV types has highly been associated with geographical regions and the extent of neoplasia in cervical cells [39, 40]. Abnormal cytology in the current study was double in cases compared to that of controls indicating how critical and essential intensification of cervical cancer screening in the HIV positive women in this region would be. Moreover, the prevalence of co-infected women with HSIL was more than threefold compared to the monoinfected. If the co-infected women were to progress to cervical cancer, then there would be a greater chance and therefore they entail a population segment that would require more screening at an earlier stage.

The overall prevalence of HR-HPV in the VIA positive women was lower than that observed by Ghedira et al [41] with women in Nigeria urban setting, a varying geographical and ethnic setting in the African context having a prevalence that was higher [42]. In current study the HR-HPV prevalence was high among women with abnormal cytology compared to normal cytology. Although the prevalence of HR-HPV in both normal and abnormal cytology in the current study are far much below those reported earlier[41] where the prevalence of HR-HPV in women with squamous intraepithelial lesions (SIL) was higher in normal cytology, the two studies are in agreement that the prevalence of HR-HPV is higher in abnormal cytology [41]. HR- HPV genotype present has been associated with the cytological abnormality with HPV 16 and 18 being associated with abnormal cytology [43]. Studies have shown that increased immune expression of p53 genes reduces HPV 16 infection rates a clear indication that differences in genetic expressions in an individual will alter the immunological responses and that will affect infectivity rate [44]. In LSIL, HPV type category (16, 18/45) had the highest prevalence followed by HPV 16 and comparative findings on HPV type 16 has been pointed also been reported in the African context [41].

In the present study, HPV type 52 has a low prevalence while type 53 was not detected. This trend is amenable with Bangkok Metropolitan women findings which indicated that the rate of HPV detection was related to severity of cytological grades with the rate following an ascending order from 13.0%, 30.8%, 39.5%, 56.3% and 100.0% for ASC-US, ASC-H, LSIL, HSIL and SCC respectively [45]. In our findings, HPV 16 prevalence increased with increase in severity of lesions ASCUS 2 LSIL 6 and HSIL 14 which was comparative to the Metropolitan findings [45]. The idea of severity of HPV cytological lesions in infection has also been reflected by the fact that abnormal cytology had a higher prevalence rate of HPV compared to normal cytology [46] which correlates to HPV genotype where HPV genotype 16 as a single infection or combined with others increase with increase of severity of lesions [47, 48].

## Conclusion

High risk HPV types 16 and 18/45 were the most predominant in the established cytological grades and among the co-infected women. There was a higher prevalence of HR-HPV genotypes in high grade lesions in co-infections which was twice that in mono-infections. Routine screening using both cytological and HPV testing should be embraced and/or reinforced as early screening and preventive strategies in the covered geographical region population. Such routine screening services would substantially mitigate progression of the pre-invasive lesions to cervical carcinoma since most women were within the low grade squamous intraepithelial ranges especially for the HIV positive. Provision of the currently available vaccines to these women at an early age would provide effective protection considering the presented findings ascertain the HPV type profiles in this population are covered by such vaccines.

### What is known about this topic

- HPV genotypes has been reported to be highly prevalent and widely varied among HPV monoinfected and HIV/HPV co-infected women in the country and beyond;
- Regional variations in the prevalence of HPV types exist in the country.

### What this study adds

- The findings provide comprehensions on genotype specific HPV trends on the most common HPV type categories in the study population in Kenya not reported before to the best of our knowledge;
- The results further provide a basis for effective screening and vaccination strategies success monitoring in the geographical setting on the basis of applying the insights in post vaccination evaluation of the control and prevention policies rolled out in Kenya on HPV vaccination.

## Competing interests

The authors declare no competing interests.
